# A re-evaluation of the scratch test for locating the liver edge

**DOI:** 10.1186/1471-230X-13-35

**Published:** 2013-02-25

**Authors:** Krishan Gupta, Akash Dhawan, Christian Abel, Nicholas Talley, John Attia

**Affiliations:** 1 John Hunter Hospital, Division of Medicine, Lookout Rd, 2305, New Lambton Heights, NSW, Australia

**Keywords:** Scratch test, Hepatomegaly, Auscultation, Liver edge, Liver span

## Abstract

**Background:**

A reliable and accurate estimation of liver size by physical examination is an important aspect of the clinical assessment of a patient. The scratch test uses auscultation to detect the lower liver edge by using the difference in sound transmission through the abdominal cavity over solid and hollow organs. The test is thought to be particularly useful if the abdomen is tense, distended, obese, or very tender. Although the sign is often taught to medical students and residents, the value of the technique for detecting the liver edge has become controversial.

**Methods:**

The study was performed in two parts. In the first part, 18 patients undergoing upper abdominal ultrasound as outpatients were randomly selected and the scratch test was performed by two raters independently, followed by ultrasound (USG) as the reference standard. In the second part of the study, the two raters independently performed the scratch test on separate randomly selected patients (15 patients by rater 1, and 16 patients by rater 2), followed by USG.

**Results:**

Agreement between raters on the scratch test was very high, with an intra-class correlation coefficient of 0.97. The agreement between the raters and the USG was 0.37 using Spearman’s rho. A Bland –Altman plot indicated that, on average, raters underestimated the distance from the right costal margin to the liver edge by only about 2.4 centimeters compared to USG. This translates into 37% and 54% of raters’ estimates falling within 2 and 3 cm of USG estimates. Each unit increase in BMI increased the discrepancy between raters and USG by 0.26 cm (p = 0.012).

**Conclusion:**

The scratch test has very high reproducibility and overall agreement between the scratch test and USG was moderate, with a spearman’s rho of 0.37. The accuracy may potentially be improved by using the point of initial sound transmission rather than the point of maximal transmission. We conclude that the scratch test deserves further investigation.

## Background

A reliable and accurate estimation of liver size by physical examination is an important aspect of the clinical assessment of a patient. The utility of various examination techniques used to determine liver size has been shown to be somewhat inconsistent, and to lack inter-observer correlation when compared with imaging methods [1-6].

The scratch test is a type of auscultatory percussion usually ascribed to Burton-Opitz in 1925 to identify the cardiac silhouette [7], although references to similar techniques date back to 1840 and have been described for various organs, including the inferior hepatic margin [8]. As applied to the liver, the scratch test uses auscultation to detect the difference in sound transmission through the abdominal cavity over solid and hollow organs. The test usually consists of placing the stethoscope below the xiphoid and lightly but briskly stroking the skin in a direction parallel to the expected liver edge, starting at the right lower quadrant and working slowly up to the right costal margin along the mid clavicular line. When the liver edge is reached, the sound of the scratch is transmitted to the stethoscope [9]. Over the years however, many variations in technique have been described [10] including:

• placement of the stethoscope near the umbilicus, the costal margin, or over the liver

• percussing with finger and pleximeter, finger alone, bristle brush, or corrugated rod

• Stroking in a circular, centripetal, centrifugal, lateral or longitudinal direction

This technique is said to be particularly useful if the abdomen is distended, obese, too tender for palpation, or if abdominal muscles are tense [9,11].

To date, only a few studies have been done to validate the reliability and accuracy of scratch test [2,12,13]. These studies have been hampered by low numbers of patients [12,13] and limited statistical analyses. Overall however, the consensus is that the test has performed poorly and recent recommendations are that the scratch test be abandoned [10,14].

We believe that this dismissal of the scratch test is premature and based on insufficient evidence. We therefore aimed to add to the evidence base by evaluating the reliability and accuracy of the scratch test to determine the lower border of liver, with comparison to ultrasound (USG) as the reference standard.

## Methods

### Design

The study was performed in two parts. In the first part, 18 patients undergoing upper abdominal ultrasound as outpatients for various indications were randomly selected; the scratch test was performed by two raters independently and followed by the ultrasound.

In the second part of the study, the two raters independently performed the scratch test on separate randomly selected outpatients (15 patients by rater 1, and 16 patients by rater 2), followed by upper abdominal ultrasound.

### Scratch test procedure

**Figure 1:**
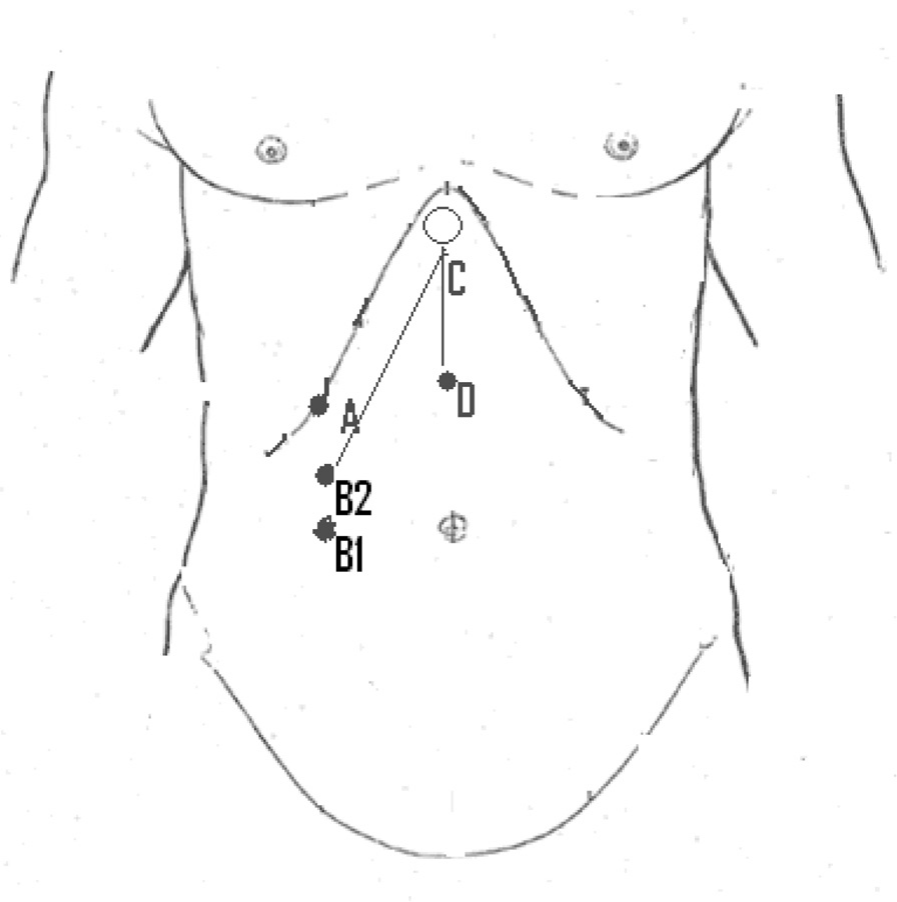
Illustration of the landmarks on the abdomen in relation to the measurements used for scratch test.

The scratch test was performed by marking a point on the right costal margin at the midclavicular line (point A in Figure 1). This point was used as a reference to take the measurements of liver span below the costal margin. The diaphragm of the stethoscope was placed on the xiphisternum (point C in Figure 1). Light transverse strokes of the skin with a single finger, parallel to the suspected liver edge, were made advancing from the right lower quadrant along the midclavicular line to the costal margin. When the hepatic edge was reached (point B1 in Figure 1), the scratching sound was transmitted through the solid liver with the resultant sudden increase in auscultated sound intensity; the sound intensity continued to increase until it was maximal (point B2) and this point was taken as the best estimate of the liver edge. The distance between this point and point A (distance AB2) was recorded on a data sheet. The sonographers used the same reference point to measure distance to the liver edge (Point A) but were blinded to the value obtained by the clinical raters. The results were recorded in centimeters between the right costal margin (RCM) and the liver edge.

The two raters (AD, KG) were senior medical registrars in the department of general medicine. Before the study, 2 calibration sessions with a consultant (JA) were performed to standardise the method, i.e. where to place the stethoscope, where to scratch, whether to listen for the point of the start of sound transmission or the point of maximal sound transmission, etc. The two raters also used the same brand of stethoscope. To ensure that the sound transmission was not purely through the skin, we measured a control point below the xiphisternum using the same stroking technique (point D in Figure 1). This point was the point of maximal sound intensity when stroked with the finger ascending upwards towards the stethoscope from below the umbilicus. The distance CD was measured in centimeters and compared with the distance B2C. If B2C was more than CD, then it was assumed that the transmission of sound heard at point B2 was through liver. AB2 was then measured as liver span below the RCM. However, if B2C was less than or equal to CD, then we assumed that the sound conduction was likely due to skin conduction and that the liver edge did not extend beyond the RCM. No clinical information about the subjects and no other methods of physical examination were performed in the study in order not to bias the interpretation of the scratch test; in particular, palpation of the liver edge was not performed.

Informed, written consent was obtained from all patients and the study was approved by the Hunter New England Area Ethics committee.

### Ultrasound procedure

The ultrasound was performed with a Phillips iU22 Ultrasound Machine (Koninklijke Philips Electronics N.V., Netherlands) using a 5-2 MHz curved array transducer with the default abdominal preset. Time Gain Compensation (TGC) curves were adjusted to optimize the image quality if required. A single focal zone was set to the mid liver parenchyma. Harmonics were off. Patients were asked to hold their breath during the ultrasound exam but not during the scratch test in order to mimic usual clinical practice.

### Statistical analysis

The co-primary outcomes were agreement between the 2 observers as measured using the intra-class correlation coefficient (ICC) and agreement between each observer and the USG reference standard as measured using Spearman’s correlation coefficient (rho), which is the non-parametric equivalent of Pearson’s coefficient.

Secondary outcomes included the degree and source of disagreement including:

•Bland-Altman plots comparing the difference between each rater and the USG (on the y-axis) compared to the USG (on the x-axis).

•the proportion of rater values that lie within 1, 2 or 3 cm of the reference value.

•whether the absolute value of the distance or the subject’s body mass index (BMI) influenced the degree of error between the clinical observer and the USG using linear regression.

Threshold p-value for significance was taken as <0.05.

## Results

A total of 49 patients were included in this study. Eighteen patients were assessed in duplicate but independently by two raters. In the second part of the study 15 patients were separately examined by the Rater 1 and 16 patients were examined by Rater 2. Characteristics of all 3 groups are given in Table 1.

**Table 1:** Characteristics of the 3 patient groups

**Demographic/characteristic**	**Class/statistic**	**Both raters (n = 18)**	**Rater 1 only (n = 15)**	**Rater 2 only (n = 16)**
Sex	Females	8 (44%)	9 (60%)	10 (62%)
	Males	10 (56%)	6 (40%)	6 (38%)
Age (years)	mean (std)	50.7 (21.3)	50.3 (24.2)	53.0 (12.0)
Height (cm)	mean (std)	166.4 (10.6)	167.5 (16.0)	165.7 (8.5)
Weight (kg)	mean (std)	71.2 (16.0)	78.9 (23.9)	75.0 (15.4)
BMI (kg/m^2^)	mean (std)	25.5 (4.3)	27.7 (6.1)	27.3 (5.5)

**Figure 2:**
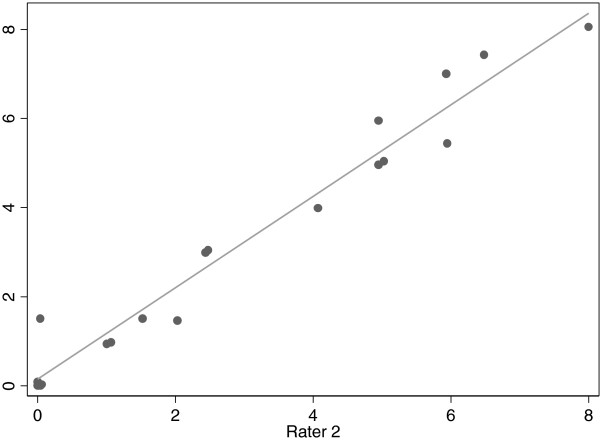
Agreement between the 2 raters on location of liver edge, measured in cm from the right costal margin.

Figure 2 shows the agreement between the 2 raters for the 18 subjects examined in duplicate. The ICC was very high at 0.97.

**Figure 3:**
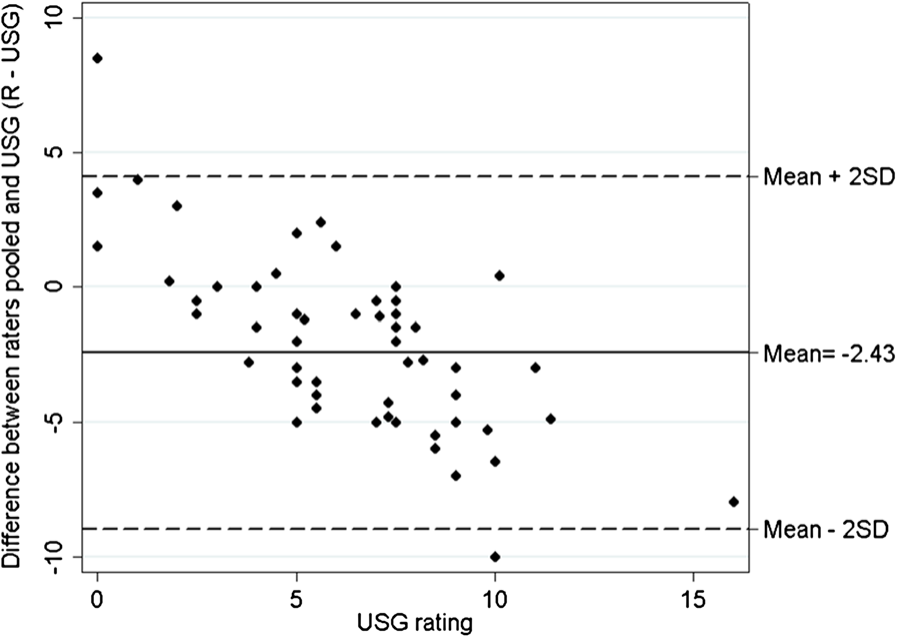
Bland-Altman plot showing difference between raters’ values and ultrasound value.

Agreement between the 2 raters and the reference USG is summarized by the Spearman’s rho of 0.37 (p-value = 0.0024) which indicates moderate agreement. The agreement between each rater and the USG is captured in a different way using the Bland Altman plot, which graphs the difference between the raters (pooled if in duplicate) and the reference standard USG (on the y-axis) vs the value of the reference standard USG (on the x-axis) (see Figure 3). This figure indicates that, on average, the raters underestimate the distance from the RCM to the liver edge by about 2.4 centimeters compared to USG.

However this difference depends to a large extent on where the liver edge lies; the closer it is to the RCM, the more the observers overestimate the distance and the farther it is from the RCM, the more the observers underestimate the distance. This average “offset” of about 2.4 cm is about the difference between the point of onset of transmission and the point of maximal transmission that was noted anecdotally during the study, i.e. difference between B1 and B2 in Figure 1.

We also calculate the proportion of ratings that are within 1, 2 or 3 cm of the reference value (Table 2, column 1). We note that 37% of ratings fall within 2 cm of the reference value and 53% fall within 3 cm. These proportions are low due partly to the average “offset” noted earlier. We extrapolate that if we used the point where transmission of the scratch started being heard (B1 in Figure 1) rather than the point of maximal transmission (B2 in Figure 1), the “offset” would be removed and these proportions would increase (see Table 2, column 2) to 43% of ratings being within 2 cm of the reference and 76% being within 3 cm.

**Table 2:** Number and percentage of ratings in each difference range compared to ultrasound (US)

**Difference with US**	**n (%)**	
	**With offset**	**Without offset**
0 – 1 cm	14 (21%)	14 (21%)
1.1 – 2 cm	11 (16%)	15 (22%)
2.1 – 3 cm	11 (16%)	22 (33%)
> 3 cm	31 (46%)	16 (24%)

The offset is 2.43 cm.

**Figure 4:**
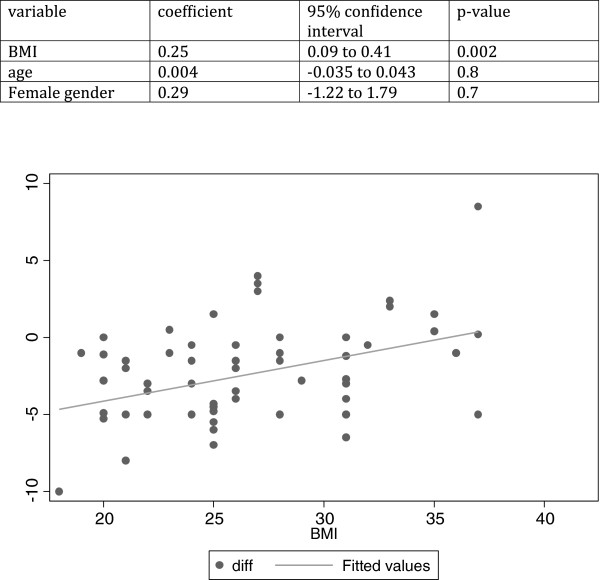
Difference between raters and USG as a function of BMI of the subject.

A linear regression indicates that BMI does significantly affect the difference between raters and the USG, with each unit increase in BMI increasing the discrepancy by 0.25cm (p=0.002). Although the most accurate ratings were at BMI of 30–35, once the “offset” of 2.4 cm is taken into account, the least difference between raters and USG is seen at a BMI of ~27. BMIs that were higher or lower than this led to overestimates and underestimates respectively of the liver span relative to the RCM (Figure 4).

## Discussion

Agreement between raters on the scratch test was very high, with an ICC of 0.97. This is much higher than previous papers; for example Joshi et al. [6] found a kappa of 0.17-0.33, and Tucker et al. [12] found a reliability coefficient of 0.68. This is likely due to the standardization sessions performed between the raters before the data collection. These however were brief (2 × 1 hour) and represent a relatively short “learning curve” compared to other physical exam maneuvers. Previous papers did not describe any standardization attempts or describe the experience of the raters with the scratch test. We speculate that this lack of standardization partly contributed to the previous estimates of poor performance for the scratch test.

We compared the scratch test vs USG for determining location of the liver edge rather than total liver span, because USG is not reliable for detecting the superior liver margin, given that this would have to be ascertained by probing intercostally and that bone interferes with ultrasound conductance. Previous studies have correlated the scratch test with overall liver span [2,12,13] and we believe that uncertainty about the location of the upper liver edge [2], as well as the lack of correlation between overall liver span and distance below the RCM [12] both contributed to poor performance of the scratch test. Furthermore, not all previous studies have made clear whether the landmark of the right costal margin at the mid-clavicular line was marked in common. Naylor et al. [15] have shown that the variation in marking this point can be up to 10 cm, and this added source of measurement error likely also contributed to the poor performance of the scratch test in previous evaluations.

The overall agreement between the scratch test and USG was moderate, with a spearman’s rho of 0.37. While this may fall well short of a perfect test (with rho of 1), the question for physical exam maneuvers is not whether they are perfect but whether they are useful enough to provide information. This correlation translates into 37% of ratings falling within 2 cm of the reference value and 53% falling within 3 cm. This is consistent with the results found by Tucker et al. [12] of 45% and 55% respectively, but falls short of the 78% accuracy within 2 cm found by Fuller et al. [13], as well as the values of 54% (within 2 cm) and 74% (within 3 cm) found by Sullivan et al. [2]. We emphasise however that both of these last 2 studies evaluated the scratch test within the context of other maneuvers and that the raters were not blinded to their own results on palpation or other percussion. By contrast, we evaluated the scratch test in isolation, with no knowledge of the history, presumed diagnosis or reason for USG, or results of palpation. While this gives a “cleaner” estimate of the performance of the scratch test, it would tend to underestimate the performance of the test.

The Bland-Altman plot, which has not been graphed by any of the previous studies, indicates an average underestimate of about 2.4 centimeters by the scratch test compared to USG. This was anecdotally the distance between the point at which sound transmission began and the point at which it was maximal (points B1 to B2 in Figure 1). We speculate that the accuracy of the scratch test may be increased by using the point at which transmission of sound begins (B1) rather than using maximal transmission (B2); this would essentially negate the “offset” of 2.4 cm and increase accuracy to 43% (within 2 cm) and 76% (within 3 cm), in line with previous studies mentioned above. This would however need to be tested prospectively.

The Bland-Altman plot also shows that raters tend to overestimate small spans and underestimate large spans. This may indicate that as the liver edge nears the RCM or the right iliac fossa, the scratch test becomes harder to perform accurately, and people estimate larger and smaller values compared to USG respectively. This bias was present despite the performance of a negative control, i.e. checking for skin transmission along the line of the umbilicus. We speculate that the bias would have been greater without this negative control given that a handful of measures (n = 5) were given a value of 0 because the measured liver span below the RCM was ascribed to skin transmission. This bias is not in keeping with previous data showing that accuracy was greater in patients with cirrhosis than in controls [1] but we speculate that this may be due to confounding by body mass index.

Indeed, we find that increasing BMI does increase the discrepancy between raters and USG. This is consistent with the study of Wolfgang et al. [16]; a sonographic survey of 2080 patients found that body mass index (BMI) and height were the most important factors affecting liver measurements at midclavicular line.

## Conclusion

Although recent textbooks suggest that the scratch test should be abandoned, we believe that the evidence base is still scanty and not sufficiently robust to rule out the usefulness of this maneuver. We find very good reproducibility between raters as well as sufficient validity compared to USG to make it useful in the physical exam armamentarium. The use of Bland-Altman plots, which has not been done in the past, suggests that the overall tendency to underestimate the distance from RCM to liver edge may be compensated by using the point of initial sound transmission rather than the point of maximal sound transmission as the indication for the liver edge. The use of a negative control by checking for skin transmission between umbilicus and xiphoid may also help increase accuracy. We conclude that the scratch test deserves further investigation.

## Abbreviations

USG: Ultrasound;RCM: Right costal margin;ICC: Intraclass correlation coefficient;BMI: Body mass index

## Competing interests

None of the authors have any conflict of interest.

## Authors’ contributions

KG, AD, and JA conceived the study. KG, AD and CA acquired the data. JA was responsible for the analysis and interpretation of the data. KG and JA drafted the manuscript. All authors critically reviewed the manuscript and approved the final version.

## Pre-publication history

The pre-publication history for this paper can be accessed here:

http://www.biomedcentral.com/1471-230X/13/35/prepub
